# Aortic Stiffness and Cardiovascular Risk in Women with Previous Gestational Diabetes Mellitus

**DOI:** 10.1371/journal.pone.0136892

**Published:** 2015-08-26

**Authors:** Tove Lekva, Jens Bollerslev, Errol R. Norwitz, Pål Aukrust, Tore Henriksen, Thor Ueland

**Affiliations:** 1 Research Institute of Internal Medicine, Oslo University Hospital, Rikshospitalet, Oslo, Norway; 2 Mother Infant Research Institute, Tufts Medical Center, Boston, Massachusetts, United States of America; 3 Section of Specialized Endocrinology, Department of Endocrinology, Oslo University Hospital, Rikshospitalet, Oslo, Norway; 4 Faculty of Medicine, University of Oslo, Oslo, Norway; 5 Department of Obstetrics & Gynecology, Tufts Medical Center and Tufts University School of Medicine, Boston, Massachusetts, United States of America; 6 Section of Clinical Immunology and Infectious Diseases, Oslo University Hospital, Rikshospitalet, Oslo, Norway; 7 Department of Obstetrics, Oslo University Hospital, Rikshospitalet, Oslo, Norway; Virgen Macarena University Hospital, School of Medicine, University of Seville, SPAIN

## Abstract

Gestational diabetes mellitus (GDM) is a significant risk factor for cardiovascular disease (CVD) in later life, but the mechanism remains unclear. The aim of the study was to investigate indices of glucose metabolism, dyslipidemia, and arterial stiffness (as measured by pulse wave velocity (PWV)), in women with and without a history of GDM, using both the old WHO and new IADPSG diagnostic criteria, at 5 years after the index pregnancy. Dyslipidemia and PWV were used as surrogate markers for CVD risk. The population-based prospective cohort included 300 women from the original STORK study. All participants had an oral glucose tolerance test (OGTT) during pregnancy. Five years later, the OGTT was repeated along with dual-energy x-ray absorptiometry, lipid analysis, and PWV analysis. Measurements were compared between those women who did and did not have GDM based on both the WHO and IADPSG criteria. We found that women with GDM based on the old WHO criteria had higher CVD risk at 5 years than those without GDM, with markedly elevated PWV and more severe dyslipidemia (higher triglycerides (TG)/HDL cholesterol ratio). After adjusting for known risk factors, the most important predictors for elevated PWV and TG/HDL-C ratio at 5-year follow-up were maternal age, BMI, GDM, systolic blood pressure, and indices of glucose metabolism in the index pregnancy. In conclusion, we found a higher risk for CVD, based on the surrogate markers PWV and TG/HDL-C ratio, at 5-year follow-up in women diagnosed with GDM in the index pregnancy when using the old WHO diagnostic criteria.

## Introduction

Gestational diabetes mellitus (GDM) refers to carbohydrate intolerance first diagnosed during pregnancy. It is a condition where pancreatic beta-cells produce inadequate amounts of insulin to meet the increased insulin needs of late pregnancy [1]. In this way, pregnancy serves as a ‘stress test’ and unmasks a preexisting predisposition to carbohydrate intolerance and decreased insulin sensitivity. A diagnosis of GDM is associated with an increased risk for maternal and fetal complications during pregnancy, and also with the woman’s lifetime risk of developing type 2 diabetes mellitus (T2DM). T2DM is a well-established independent risk factor for cardiovascular disease (CVD) [2], and it has therefore been suggested that women with a history of GDM may also be at increased risk of developing CVD. However, only a few large population-based retrospective studies have investigated the association between prior GDM and long-term adverse CV outcome [3,4]. The early identification of modifiable surrogate risk markers that may predict future CVD risk is critically important for risk stratification and for the development of strategies for primary prevention, and such markers could also be used to study the effect of GDM and CV risk during follow-up.

Diabetic dyslipidemia with hypertriglyceridemia, reduced high-density lipoprotein (HDL) cholesterol concentrations, and a shift towards small dense low density lipoprotein (LDL) is thought to be responsible for the increased CV risk in T2DM patients and can be detected many years before the clinical diagnosis of this disorder [5]. Increased HOMA-IR is believed to be the main trigger for diabetic dyslipidemia and may influence vascular function by several mechanisms such as insulin-mediated proliferation of vascular smooth muscle cells and lipid synthesis, with subsequent LDL binding and extracellular matrix remodeling within the vessel wall leading to enhanced vascular stiffness [6,7]. High pulse wave velocity (PWV) reflects increased arterial stiffness, and carotid-femoral PWV (aortic PWV) is considered the gold standard for measuring arterial stiffness [8], and increased arterial stiffness is an independent risk factor for adverse CV outcome in the general population [9]. Arterial stiffness is an age related process and is accelerated in T2DM [10]. It is also increased in pre-diabetic states and may be used to predict the onset of T2DM [11]. However, there are limited long-term data on arterial stiffness in women with previous GDM.

The GDM diagnostic criteria have changed over the years. New criteria have been proposed based on the HAPO study, which primary focus was to identify women at risk for delivering a large-for-gestational-age infant [12], and attention was to identify women at high risk of short-term adverse perinatal outcomes and not long-term maternal outcomes [13]. The WHO itself has called for additional research to better understand the ability of the new GDM criteria to predict long-term maternal adverse outcomes [13]. To this end, we have conducted a 5-year follow-up of 300 women originally recruited into the prospective STORK cohort that followed 1031 low-risk Norwegian women throughout pregnancy [14]. The primary aims of the current study are: (1) to investigate surrogate markers of CVD risk (specifically PWV and dyslipidemia) at 5-year follow-up in women who did and did not have GDM in their index pregnancy as defined by both the old WHO and the new IADPSG diagnostic criteria; and (2) to evaluate the associations between more detailed indices of glucose metabolism measured during the index pregnancy and subsequent glycemic control, lipid parameters and PWV at 5-year follow-up.

## Materials and Methods

### Study population

#### Pregnancy

The STORK study was a prospective cohort study with a longitudinal design in which 1031 low-risk women of Scandinavian heritage who gave birth at Oslo University Hospital Rikshospitalet between 2002 and 2008 were followed throughout their pregnancy. Details about the study have been previously published [14]. Briefly, each pregnant woman had four antenatal visits at weeks 14–16, 22–24, 30–32, and 36–38. A 75g oral glucose tolerance test (OGTT) was performed on all women at 30–32 weeks of gestation.

#### Follow-up

The current study is a 5-year follow-up after the index pregnancy [15]. A total of 1031 participants from the original STORK cohort were invited to participate; the 10 women who developed preeclampsia in the index pregnancy (including 2 who developed both GDM and preeclampsia) were not included in this paper to avoid the obvious confounder of preeclampsia. There were 9 women who delivered preterm, between 34–37 week, and these women was not excluded from this paper. Exclusion criteria included pregnancy at the time of invitation and delivery within the past year. Three hundred women agreed to participate. Written informed consent was obtained from all participants in the study. All clinical investigations were conducted according to the principles in the Declaration of Helsinki. The study was approved by the Regional Committee for Medical Research Ethics of Southern Norway in Oslo, Norway.

At the time of the 5-year follow-up visit, a fasting blood draw was performed to measure lipid profiles and a 75g OGTT was conducted.

### Measurements of glycemic and lipid parameters

#### Pregnancy

All 75g OGTTs were performed in the morning after an overnight fast (minimum 8 hours). Venous EDTA blood was analyzed at point of care using an Accu-Check Sensor glucometer (Roche Diagnostics GmbH, Mannheim, Germany). Additional venous blood samples were allowed to clot for 30 minutes and the serum separated by centrifugation for 10 minutes at 3000*g* and stored at -80°C. Glucose levels were also measured from frozen serum samples collected at 30–32 weeks using the hexokinase method (Hitachi Modular P800, Roche Diagnostics, Mannheim, Germany) at an accredited clinical chemistry laboratory at Oslo University Hospital Rikshospitalet, as previously reported [15].

#### Follow-up

For the 5-year follow-up study, we used the glucose data from the Accu-check Sensor glucometer (Roche Diagnostics, Mannheim, Germany). Insulin levels in the stored samples were assayed in duplicate by RIA (Diagnostic Products Corporation, Los Angeles, CA), as previously reported [15]. Levels of apolipoprotein (apo)A, apoB, HDL cholesterol (C), LDL-C (directly measurements), and triglycerides (TG) were measured from frozen serum samples at follow-up at an accredited clinical chemistry laboratory at Oslo University Hospital Rikshospitalet. The ratios of apoB/apoA, HDL/LDL-C and TG/HDL-C are shown to be risk factors for CVD [16,17], and were calculated based on the above measurement.

### Diagnosis of GDM

#### Pregnancy

GDM was diagnosed on a 75g OGTT using both the new IADPSG criteria and the old WHO criteria as follows: (1) IADPSG criteria: fasting plasma glucose (FPG) 5.1–6.9 mmol/L) and 1h plasma glucose ≥10.0 mmol/L or 2 hours plasma glucose 8.5–11.0 mmol/L; and (2) WHO criteria: 2 hours plasma glucose ≥7.8 mmol/L [13].

#### Pregnancy and follow-up

Insulin sensitivity, beta-cell function and HOMA-IR were calculated as previously reported [15]. Insulin sensitivity was measured on the same samples collected at the time of OGTT using the Matsuda index (i.e., 10,000 / square root of [fasting glucose (mmol/L) x fasting insulin (mU/L)] x [mean glucose (mmol/L) x mean insulin (mU/L)]) during OGTT. This index is a measure of whole body insulin sensitivity that has been validated against the euglycemic-hyperinsulinemic clamp [18]. beta-cell function was assessed with the insulin secretion-sensitivity index (ISSI-2) (area under the curve (AUC) insulin (mU/L) _0–120_ / glucose (mmol/L) _0–120_ x Matsuda), which has been validated against the disposition index from the intravenous GTT [19]. Homeostasis model assessment: insulin resistance (HOMA–IR) was calculated as fasting insulin (mU/L) × fasting glucose (mmol/L) / 22.5, as previously described by Matthews et al [20].

### Measurements of arterial stiffness

#### Follow-up

All participants were examined at the 5-year follow-up visit on the morning after fasting overnight. Aortic stiffness was assessed by means of PWV measurements using SphygmoCor (Atcor Medical, Sydney, Australia), a non-invasive technique with direct-contact pulse sensors. Aortic PWV was measured by sequential recordings of the arterial pressure waveform at the carotid and femoral arteries. The PWV was calculated as the distance between recording sites (suprasternal notch to femoral) measured over the surface of the abdomen (*L*), divided by the time interval (*t*) between the feet of the flow waves (PWV  =  *L*/*t*). The value was averaged over 10 cardiac cycles [21]. Only measurements that met the automatic quality control cutoff were used in the final analysis. Average SD of all measurements (mean time difference between carotid and femoral) was below 5%. All measurements were performed by the same technician.

### Measurements of body fat composition

#### Follow-up

Total body composition was determined by dual-energy X-ray absorptiometry (DXA; GE Lunar Prodigy Densitometer (software version 12.10), GE Medical Systems, Lunar Corp., Madison, WI, USA) and analyzed using enCORE software (version 14.10; GE Medical Systems), as previously described ([15] et al, accepted EJE) and. All DXA scans were performed by a single technician. CoreScan has been previously validated against volumetric computed tomography [22,23]. For measuring android fat, a region of interest (ROI) was defined with the caudal limit at the top of the iliac crest and the cephalic limit at the base of the skull. Android ROI contains both visceral (VAT) and subcutaneous adipose tissue (SAT). The software estimates the quantity of SAT in the android ROI. VAT was computed by subtracting SAT from the total android fat. The fat mass data from DXA was transformed to volume using a constant correction factor (0.94g/cm^3^) consistent with the density of adipose tissue [22]. All VAT under 50g was set to 50g since the DXA measurement is unreliable in the low range visceral fat content [24].

### Statistical analysis

Statistical analyses were conducted using SPSS for Windows, version 21.0 (Chicago, IL, USA). Data are expressed as mean±SD when normally distributed and median (25^th^, 75^th^ percentile) when skewed. Comparison between women with and without a history of GDM was performed using t-test or Mann-Whitney U depending on distribution, and Chi-square test for categorical variables. Univariate and stepwise (probability of F to-enter 0.1-remove 0.15) linear regression analyses were carried out on log transformed variables (if skewed) and results given as standardized regression coefficients. Variables below p<0.2 were included in the stepwise multivariable models. Receiver operating characteristic (ROC) curves of the glucose metabolism markers were created to identify whether a parameter was a significant predictor of PWV and TG/HDL-C ratio, and the AUC was used to evaluate the predictive efficiency of each parameter. Values of 0.9–1 indicate excellent predictive accuracy, values 0.8–0.9 good accuracy, values 0.7–0.8 fair accuracy, values 0.6–0.7 poor accuracy, and 0.5–0.6 unacceptably poor accuracy [25,26]. Interactions between GDM, body mass index (BMI, in kg/m^2^), blood pressure (BP), and indices of glucose metabolism were evaluated by univariate general linear model or linear regression with TG/HDL-C ratio as the dependent variable and indices of glucose metabolism, GDM, and the interaction term as the independent variables. Based on the averages (between 6.0 and 6.5) and standard deviations (~0.8) from the study by Heitritter et al. [27] and an incidence of GDM between 10–15% (depending on criteria) we considered that a 5% difference could be clinically significant (based on the small SDs and young age of the women). Thus, around 30% participation from the original study (n~300) would be needed to yield a statistical power around 80%. Two-tailed p-values <0.05 were considered significant.

## Results

Table 1 shows the characteristics of the study population during the index pregnancy and at the time of the 5-year follow-up visit stratified into those women who did and did not have GDM in the index pregnancy using both the IADPSG (50 with and 234 without GDM) and WHO criteria (31 with and 253 without GDM), based on complete OGTTs. As evidenced from Table 1, women with GDM based on the IADPSG criteria were on average about 1 year older, had a higher BMI both in the index pregnancy and at follow-up, had more large for gestational age (LGA) infants, and were more frequently smokers at follow-up compared to the non-GDM group. Women with GDM based on the WHO criteria also had a higher BMI at follow-up compared to their non-GDM counterparts, lower gestational weight gain in pregnancy, and also more frequently smokers at follow-up. Preterm birth could potentially influence our results, and after excluding these, only the smoking results were changed and were now not significant between GDM and non- GDM, except previous smoking at follow-up in the IADPSG criteria between GDM and non GDM (p = 0.046).

**Table 1:** Characteristics of the study population according to the new GDM IADPSG criteria and the old GDM WHO criteria.

		Visit 3 (week 30–32) in the index pregnancy		Follow-up visit	
Variable		GDM	Non-GDM	GDM	Non-GDM
N =	IADPSG	50	234	50	234
	WHO	31	253	31	253
Follow-up time (years)	IADPSG			5.1 (4.6, 5.3)	4.8 (4.4, 5.4)
	WHO			5.0 (4.5, 5.4)	4.8 (4.4, 5.4)
Age (years)^1^	IADPSG	33.6±4.3	32.0±3.7*	38.9±4.4	37.4±3.7*
	WHO	33.1±3.7	32.2±3.8	38.6±3.8	37.5±3.8
Height (cm)^1^	IADPSG	169±6	169±6	168 ±6	169±6
	WHO	168±5	169±6	168±5	169±6
BMI (kg/m2)	IADPSG	28.2 (26.8, 30.8)	26.2 (23.7, 28.4)**	24.7 (22.5, 28.0)	22.6 (20.8, 24.6)**
	WHO	27.8 (25.7, 31.2)	26.4 (23.9, 28.6)	24.1 (21.7, 28.1)	22.8 (20.9, 25.1)*
Primipara n (%)	IADPSG	n (44.0%)	n (51.1%)	n (12.0%)	n (11.1%)
	WHO	n (60.0%)	n (48.6%)	n (19.3%)	n (10.3%)
Gestational weight gain (kg)	IADPSG	9.8 (8.3, 12.1)	10.1 (8.1, 12.6)		
	WHO	9.4 (6.1, 11.4)	10.3 (8.3, 12.9)*		
Gestational age at delivery (weeks)	IADPSG	40.4 (39.0, 41.3)	40.4 (39.3, 41.1)		
	WHO	40.3 (39.0, 40.7)	40.4 (39.3, 41.1)		
Birth weight (g)	IADPSG	3832±530	3588±502*		
	WHO	3740±455	3640±520		
SGA/LGA (%)^2^	IADPSG	n (2.0/28%)	N (1.7/12.4%)*		
	WHO	n (0/16.1%)	n (2.0/15.0%)		
Preterm (n)^3^	IADPSG	3	6		
	WHO	2	7		
Family history heart disease n (%)	IADPSG			n (64.5%)	n (57.7%)
	WHO			n (75.9%)	n (57.0%)
Family history diabetes n (%)	IADPSG			n (34.0%)	n (30.3%)
	WHO			n (41.9%)	n (29.6%)
Currently smoking n (%)	IADPSG	n (2.0%)	n (3.0%)	n (26.0%)	n (15.0%)*
	WHO	n (3.2%)	n (2.8%)	n (29.0%)	n (15.4%)*
Previous smoker n (%)	IADPSG	n (28.0%)	n (16.7%)	n (30.0%)	n (21.3%)
	WHO	n (25.8%)	n (17.8%)	n (25.8%)	n (22.5%)
Systolic blood pressure (mmHg)	IADPSG	115 (105, 120)	110 (105, 120)	110 (100, 130)	110 (100, 120)
	WHO	110 (100, 120)	110 (105, 120)	110 (100, 130)	110 (100, 120)
Diastolic blood pressure (mmHg)	IADPSG	70 (60,73)	70 (60, 70)	70 (65, 75)	70 (60, 75)
	WHO	70 (60, 70)	70 (60, 70)	70 (65, 80)	70 (60, 75)
Mean arterial pressure (mmHg)	IADPSG	83.3 (78.3, 88.3)	81.7 (76.7, 86.7)	83.3 (76.7, 92.1)	83.3 (76.7,88,3)
	WHO	82.5 (77.5, 86.7)	83.3 (76.7, 86.7)	83.3 (78.7, 95.0)	83.3 (76.7, 88.3)
Pulse pressure (mmHg)	IADPSG	45 (40,50)	43 (40,50)	40 (40,50)	40 (40,50)
	WHO	40 (40,50)	45 (40,50)	40 (35,50)	40 (40,50)

^1^ Visit 1 (week 14–16 in the index pregnancy).

^2^ SGA (small for gestational age) <2500, LGA (large for gestational age)>4200

^3^ Born between 34–37 weeks

Data are given as mean±SD when normally distributed and median (25^th^, 75^th^) when the distribution was skewed. Comparison between women with GDM and non-GDM were performed using t-test for normally distributed variables, Mann-Whitney U for non-distributed continuous variables, and Chi” test for categorical variables.

* p<0.05

** p<0.001

### Lipids in women with previous GDM at 5-year follow-up

Using the IADPSG GDM criteria, we found that GDM women had significantly decreased apoA levels and HDL/LDL-C ratios and significantly increased TG levels and apoB/apoA and TG/HDL-C-ratios compared to non-GDM women at follow-up. However, after adjusting for age, BMI, and frequency of smokers (current and history), there were no significant difference between the GDM and non-GDM women (Table 2).

**Table 2:** Pulse wave velocity and lipids between GDM and non-GDM based on IADPSG and WHO criteria.

	IADPSG criteria				WHO criteria			
Variable	GDM (n = 50)	Non-GDM (n = 234)	Crude p-value	Adjusted p-value^1^	GDM (n = 31)	Non-GDM (n = 253)	Crude p-value	Adjusted p-value^1^
PWV (m/s)	6.7 (6.4, 7.3)	6.6 (6.1, 7.1)	0.050	0.270	6.9 (6.4, 7.3)	6.6 (6.1, 7.1)	**0.013**	**0.046**
ApoA (g/L)	1.50 (1.34, 1.69)	1.55 (1.40, 1.77)	**0.043**	0.117	1.39 (1.27, 1.56)	1,55 (1.40, 1.77)	**<0.001**	**<0.001**
ApoB (g/L)	0.72 (0.58, 0.86)	0.69 (0.58, 0.80)	0.070	0.388	0.75 (0.61, 0.85)	0.69 (0.58, 0.80)	0.159	0.350
HDL-C (mmol/L)	1.40 (1.20, 1.73)	1.54 (1.36, 1.82)	**0.007**	0.058	1.30 (1.08, 1.52)	1.54 (1.36, 1.83)	**<0.001**	**<0.001**
LDL-C (mmol/L)	2.66 (2.15, 3.20)	2.50 (2.09, 3.00)	0.123	0.405	2.61 (2.10, 3.11)	2.52 (2.10, 3.02)	0.614	0.909
TG (mmol/L)	0.78 (0.66, 0.95)	0.72 (0.58, 0.91)	**0.012**	0.261	0.87 (0.67, 1.17)	0.73 (0.58, 0.91)	**0.001**	**0.004**
ApoB/apoA ratio	0.48 (0.40, 0.61)	0.45 (0.36, 0.53)	**0.009**	0.109	0.53 (0.42, 0.62)	0.44 (0.36, 0.53)	**0.002**	**0.006**
HDL/LDL-C ratio	0.54 (0.41, 0.72)	0.61 (0.50, 0.82)	**0.005**	0.074	0.50 (0.40, 0.62)	0.61 (0.49, 0.81)	**0.002**	**0.008**
TG/HDL-C ratio	0.52 (0.42, 0.76)	0.45 (0.36, 0.63)	**0.001**	0.065	0.65 (0.45, 1.03)	0.45 (0.36, 0.62)	**<0.001**	**<0.001**

^**1**^ Adjusted for age, BMI, and frequency of current and previous smokers.

Data are given as median (25^th^, 75^th^).

Comparison between women with GDM and non-GDM were performed using univariate general linear model on log transformed data.

For the WHO criteria group, we found that the GDM women had significantly decreased levels of apoA and HDL-C and decreased HDL/LDL-C ratios as well as significantly increased TG measurements and increased apoB/apoA and TG/HDL-C ratios compared to non-GDM women at follow-up. After adjusting for age, BMI and frequency of smokers, the differences in apoA, HDL-C, TG, apoB/apoA ratio, HDL/LDL-C ratio, and TG/HDL-C ratio between the GDM and non-GDM groups remained significant (Table 2).

### Pulse wave velocity in women with previous GDM at 5-year follow-up

For the WHO criteria group, we found significantly increased arterial stiffness as assessed by PWV measurements and this was seen also after adjusting for age, BMI and frequency of smokers (Table 2). In contrast, no such association was seen when using the IADPSG GDM criteria. (Table 2)

### GDM, using the WHO diagnostic criteria, is a predictor of the CV risk factors PWV and TG/HDL-C in multivariable analysis at 5-year follow-up

An increased TG/HDL-C ratio has been identified as a risk factor for CVD in hypertensive populations [17]. Our findings so far show that women with GDM based on the WHO diagnostic criteria (but not the IADPSG diagnostic criteria) had stronger CVD risk at 5-year follow-up, based on elevated PWV and TG/HDL-C ratios (Table 2). For this reason, we chose to focus our subsequent analyses on those women with GDM identified by the WHO criteria only. As shown in Table 3, significant associations were found between PWV, age, GDM, systolic BP, diastolic BP, and visceral fat volume at follow-up in univariate analysis. In stepwise multivariable regression, age, GDM, and systolic BP were found to be significant predictors of PWV at 5-year follow-up. Furthermore, the TG/HDL-C ratio correlated with BMI, smoking (current and history), GDM, systolic BP, diastolic BP and visceral fat volume at follow-up. In stepwise multivariable regression, BMI, GDM, and systolic BP were found to be the strongest predictors of TG/HDL-C ratio at 5-year follow-up.

**Table 3:** Predictors of PWV and TG/HDL ratio at 5-year follow-up based on GDM status in the index pregnancy using the WHO diagnostic criteria.

	PWV				TG/HDL-C ratio			
	Univariate		Multivariable		Univariate		Multivariable	
Variables	r	p	β	p	r	p	β	p
Age (years)	0.23	**<0.001**	0.21	**<0.001**	0.01	0.906		
Follow-up time (years)	0.01	0.963			0.01	0.916		
BMI (kg/m^2^)	0.08	0.191			0.42	**<0.001**	0.32	**<0.001**
Parity^1^	0.01	0.943			-0.06	0.337		
Smoking^2^	0.10	0.108			0.15	**0.013**	0.10	0.063
Family history of diabetes	0.01	0.884			0.11	0.071		
Family history of heart disease	0.03	0.570			0.033	0.577		
GDM (WHO criteria)	0.14	**0.018**	0.13	**0.025**	0.28	**<0.001**	0.24	**<0.001**
Systolic BP (mmHg)	0.27	**<0.001**	0.28	**<0.001**	0.26	**<0.001**	0.24	**0.002**
Diastolic BP (mmHg)	0.21	**<0.001**			0.12	**0.044**	-0.14	0.063
Visceral fat volume (g/cm^3^)	0.15	**0.012**			0.42	**<0.001**		
R square				0.14				0.26

^**1**^ Defined as primiparous (for the purposes of the 5-year follow-up)

^**2**^ Refers to both current and previous smokers

### Prediction of PWV and TG/HDL-C ratio at follow-up by indices of glucose metabolism during the index pregnancy

Since, in our cohort, GDM diagnosed by the WHO criteria was associated with enhanced CV risk at 5-years as determined by an increased PWV and TG/HDL-C ratio, we further evaluated the association between these measures and indices of glucose metabolism (i) during the index pregnancy and (ii) at follow-up. To define women at high risk of CV disease at follow-up, we used a PWV value of greater than the 90^th^ percentile and a TG/HDL-C ratio of >1.09, a cut-off previously used to identify CVD in hypertensive patients [17].

**Fig 1:**
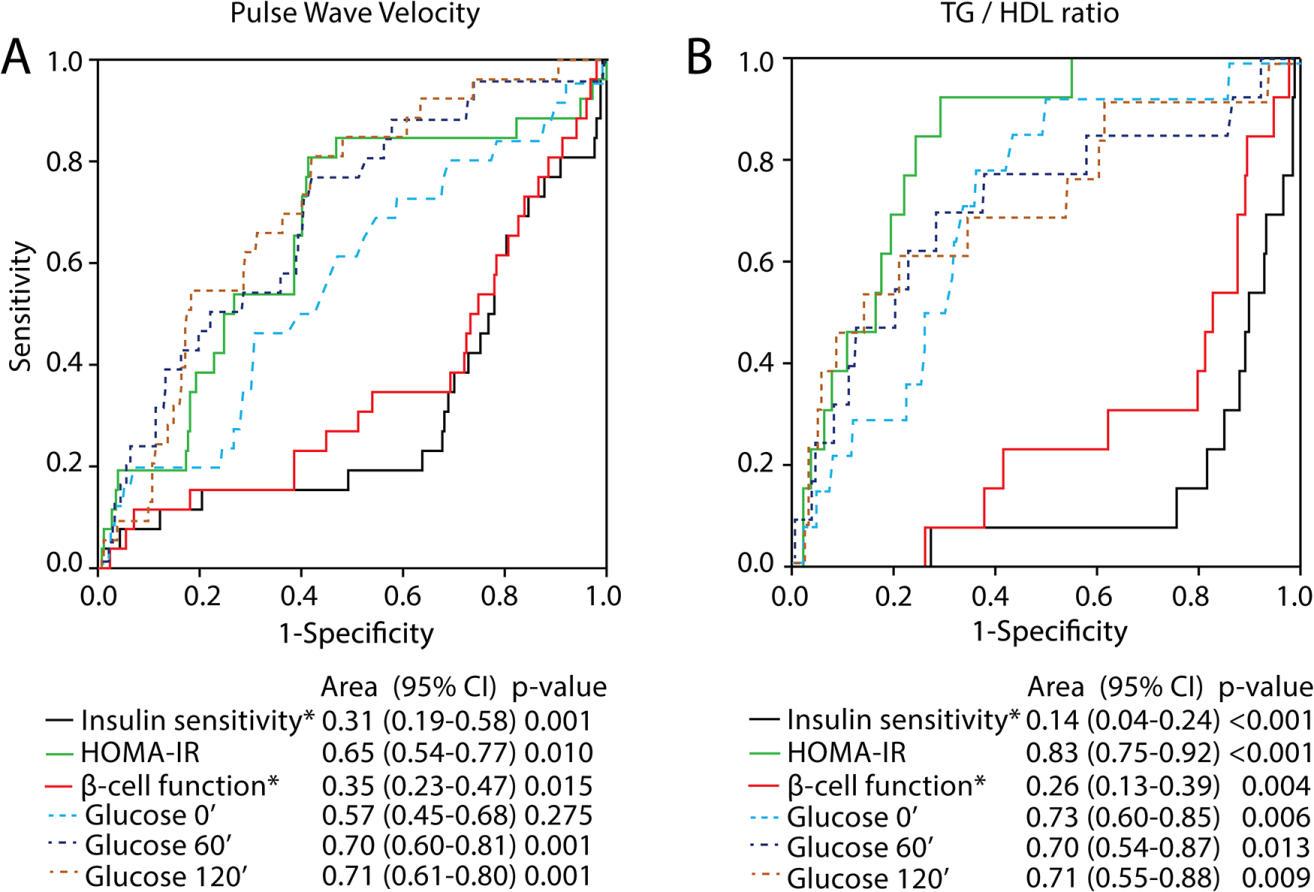
Glucose tolerance in late pregnancy is associated with an elevated PWV and TG/HDL-C ratio at 5-year follow-up. Receiver operating characteristic (ROC) curves for predicting (A) PWV and (B) TG/HDL-C ratio by HOMA-IR (green line), insulin sensitivity (black line), beta-cell function (red line), fasting glucose (light blue dotted line), glucose 60 min (dark blue dotted line) glucose 120 min (brown dotted line) in pregnancy. *Risk is expressed as 1-AUC for beta-cell function and insulin sensitivity for comparison reasons.

As shown in Fig 1, receiver operating characteristic analysis indicated that glucose levels during the 75g OGTT and HOMA-IR, insulin sensitivity, and beta-cell function measured at 30–32 weeks in the index pregnancy had a poor to fair accuracy for the prediction of an elevated PWV at 5-year follow-up. For the TG/HDL-C ratio at follow-up, measurements of insulin sensitivity and resistance at 30–32 weeks displayed a good accuracy for prediction.

### Interactions between indices of glucose metabolism after 5 years follow-up and GDM risk factors on PWV and the TG/HDL-C ratio

**Fig 2:**
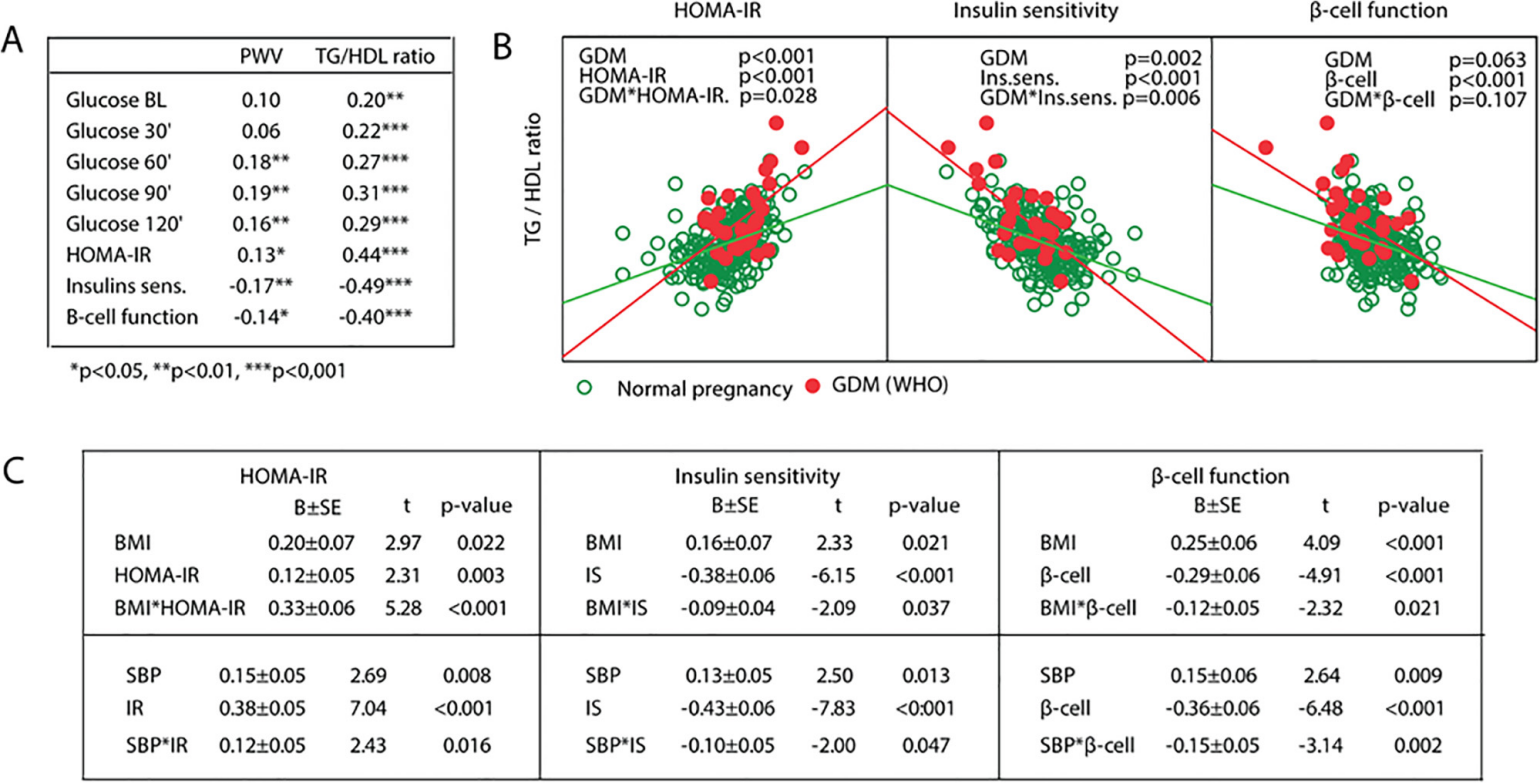
Relationship between measurements of glycemic control and vascular stiffness at 5-year follow-up in patients with (red circles) and without GDM (green circles) in the index pregnancy. (A) Associations between PWV, TG/HDL-C ratio and glucose levels during OGTT, HOMA-IR, insulin sensitivity and beta-cell function in the whole cohort at follow-up. (B) Interaction analysis between GDM (red) and indices of glucose metabolism on the TG/HDL ratio. (C) Interaction analysis between BMI and SBP and indices of glucose metabolism on the TG/HDL ratio.

When investigating the associations between measurements of glucose metabolism and PWV and TG/HDL-C ratio at 5-year follow-up, we found moderate but significant positive associations between PWV and the later time points of the OGTT (60 minutes and 120 minutes) and HOMA-IR, while insulin sensitivity and beta-cell function were negatively correlated with PWV (Fig 2A). Similarly, glucose levels during the OGTT were moderately correlated with the TG/HDL-C ratio with stronger associations towards the end of the test (Fig 2A). Even stronger associations were observed between the TG/HDL-C ratio and indices of glucose metabolism, with a positive correlation with HOMA-IR and negative correlations with insulin sensitivity and beta-cell function.

To evaluate whether the associations between the indices of glucose metabolism and CV risk measurements were stronger in women with previous GDM, we performed an interaction analysis. As shown in Fig 2B, we found a significant interaction between GDM and both HOMA-IR (p = 0.028) and insulin sensitivity (p = 0.006) (but not beta-cell function) at 5-year follow-up for the TG/HDL-C ratio, but not PWV (data not shown). These data suggest that other risk factors present in women with GDM may enhance the adverse effects of glucose intolerance on the TG/HDL ratio. Additional interaction analyses were performed using BMI and systolic BP instead of GDM, and these gave similar results (Fig 2C). Excluding the women with preterm birth changed our results for Fig 2C and the interaction between SBP and insulin sensitivity were now not significant (p = 0.071).

## Discussion

The current study report 5-yr longitudinal CVD risk assessment data from women with and without a history of GDM (as per WHO vs. IADPSG criteria). Largely the aim was to assess the 5-yr predictability of glucose tolerance measures during pregnancy on CVD risk factors, mainly dyslipidemia (TG/HDL ratio) and arterial stiffness (PWV). The most salient findings were the following: 1) Women with GDM as diagnosed by WHO criteria had a higher risk for CVD 5 years after the index pregnancy; 2) Having GDM (WHO criteria) strongly predicted arterial stiffness and a high TG/HDL ratio. Moreover, there was a strong interaction between GDM and SBP in the prediction of PWV, and with SBP and BMI to predict TG/HDL ratio; 3) The 1- hour and 2-hour post 75g glucose load measures strongly predicted PWV (by ROC), while HOMA-IR most strongly predicted TG/HDL ratio (ROC). Overall, these data add important insight to the evolution of CVD risk in women with GDM.

A few small case-control studies have investigated arterial stiffness in women with GDM in late pregnancy and have shown no significant effect on PWV [28–30]. Similarly, there appears to be no effect of GDM or hyperglycemia in the immediate postpartum period (i.e., within 2 months of delivery) on arterial stiffness [31]. Taken together, these studies suggest that short-term exposure to hyperglycemia may be insufficient to increase arterial stiffness. Using a retrospective case-control approach, Heitritter et al. examined 48 healthy women of which 25 had a history of GDM an average of 4–5 years after pregnancy and detected no difference in PWV [27]. In contrast, in this population-based prospective cohort study of 284 women of which 31 had GDM we observed a significant increase in PWV at a similar time-point in women with GDM based on the old WHO diagnostic criteria (Table 2). This difference could be explained by our prospective study design (and therefore a larger non-GDM group) and standardized follow-up time for all participants. Interestingly, their study did demonstrate evidence of early vascular dysfunction using other hemodynamic parameters (i.e., increased peripheral vascular resistance) [27]. Our findings suggesting the presence of early vascular dysfunction in previous GDM women are further supported by reports showing and altered endothelial function [32,33] and higher common carotid artery intima-media thickness 6 years postpartum in previous GDM women [34]. In addition to GDM, the current study also identified using multivariable analysis a number of well known risk factors of CVD, such as age and systolic BP, as significant predictors of an increased PWV [10]. Of note, we did not find a significant association between BMI and PWV. The impact of BMI on arterial stiffness is debated [35]. The absence of an association in the current study could partly be explained by the fact that these were relatively healthy Scandinavian women most of whom had a normal BMI. Nonetheless, the univariate association between PWV and VAT mass may suggest some effects of excess adipose tissue on early changes in arterial stiffness.

Our finding of dyslipidemia in women with GDM at 5-year follow-up, which was more pronounced based on the old WHO diagnostic criteria for GDM, and in women with a more severe glucose intolerance during pregnancy, are consistent with a number of other studies demonstrating an atherogenic lipid profile in such women [36,37]. In particular, elevated TG levels are common in dyslipidemia that accompanies the pre-diabetic state and are closely correlated with enhanced CV risk [38]. Recently, the TG/HDL-C ratio has been identified as a surrogate cardio-metabolic risk marker that may predict adverse CV outcome in hypertensive subjects (22). Sokup et al. recently reported that TG, HDL-C, and the TG/HDL-C ratio were elevated in GDM women at around one year after the index pregnancy, and may represent an early marker of endothelial dysfunction and CV risk [39]. They further found that this atherogenic lipid profile in non-diabetic women with a history of GDM was independent of both HOMA-IR and BMI. In contrast, we found that the TG/HDL-C ratio was strongly associated with a diagnosis of GDM and associated risk factors, including BMI and systolic BP. Furthermore, when evaluating associations between indices of glucose metabolism and the TG/HDL-C ratio, we found a robust correlation with integrated measures of glucose intolerance. Indeed, the interactions we demonstrate between a diagnosis of GDM risk factors and HOMA-IR and sensitivity on the TG/HDL-C ratio further suggest that underlying risk factors in GDM may enhance the adverse effects of glucose intolerance on CV risk. The interaction analysis with both BMI and systolic BP showing similar results further support this assertion. Taken together, our data support a recent large population based case-control study demonstrating that GDM is a significant CVD risk factor. Our data also suggest that a combination of GDM diagnosis with other risk factors (BMI in particular) could identify individuals at particularly enhanced CVD risk [40]. Our data indicate that the underlying mechanism responsible for this increased CVD risk may be dyslipidemia with an unfavorable pro-atherogenic balance between HDL-C and TG.

The long-term risk of CVD following a pregnancy complicated by GDM as defined by the old WHO criteria compared to the new IADPSG criteria has not been systematically examined. Our findings suggest that women classified with GDM by the old WHO criteria have a higher risk of future CVD as estimated by increased arterial stiffness and more pronounced dyslipidemia a median of 4.8 years following pregnancy. No such effect was seen in women diagnosed with GDM using the new IADPSG criteria. This suggests that CV risk may be underestimated or identified at a later time-point using the new IADPSG criteria, which could impact early use of preventive strategies. The main difference between the old WHO and the new IADPSG criteria is the lowering of fasting plasma glucose and inclusion of a 1 hour glucose cutoff in the criteria. It should be mentioned that populations with different ethnicity have various glucose abnormalities, for instance high frequency of elevations in fasting glucose or post-load glucose, and use of the IADPSG criteria in different populations could yield different outcomes [41]. Finally, Retnakaran and Shah performed a large retrospective population-based cohort study of 435,696 women and found that, even in the absence of GDM, women who have mild glucose intolerance in pregnancy (i.e., those with an abnormal 1-hour glucose challenge test but a normal OGTT) may be at increased risk of CVD [42].

Strengths of this study include the fact that it is a well characterized population-based cohort with standardized measurements of PWV and lipid profiles and a similar follow-up time between the GDM and non-GDM groups. Moreover, the women were generally young and healthy, which makes the observed differences in PWV and lipid profiles even more significant. Such differences may be even more dramatic in older women and those with underlying co-morbid medical conditions. An additional strength of this study is that the investigators have used IADPSG criteria to identify maternal risk—and the criteria found less incidence of risk vs. WHO. Limitations are the lack of more robust evidence of CVD such as intima media thickness and number of women with GDM was relatively low. Power analysis suggested we were at the low end for identifying significant differences in PWV and with a larger cohort, PWV might have remained significantly difference between the groups in adjusted analysis also. PWV measurement may not be optimal if the abdominal fat volume is large, and is also a limitation to the PWV analysis. However, the women in our study have mostly normal BMI.

In summary, our data showing enhanced CV risk at 5-year follow-up as reflected by increased arterial stiffness (elevated PWV) and elevated TG/HDL-C ratio with increased glucose intolerance in late pregnancy as diagnosed using the old WHO criteria support the conclusion that important information on CV risk may be gained by antepartum glucose tolerance screening. Exactly which measures give the most information and whether an increase in PWV and/or TG/HDL-C ratio actually translates into an increased incidence of CVD and adverse CV outcome in women who experience GDM must be evaluated in large population-based studies.
